# Correlation study between multiplanar reconstruction trigeminal nerve angulation and trigeminal neuralgia

**DOI:** 10.1186/s12883-022-02906-9

**Published:** 2022-10-12

**Authors:** Tao Sun, Qinghao Huang, Chuangfeng Li, Wensheng Yang, Wentao Wang, Longshuang He, Jinlong Liu, Chao Yang

**Affiliations:** 1 Department of Neurosurgery, First Affiliated Hospital of Sun Yat-Sen University, No 58th, Zhongshan Er Road, Yuexiu District, Guangzhou, 510080 China; 2 Center of Universal Medical Imaging Diagnostic, No 80th, Zhongshan Er Road, Yuexiu District, Guangzhou, 510080 China; 3 Department of Neurosurgery, First Affiliated Hospital of Guangdong Pharmaceutical University, No. 19, Nonglinxia Road, Yuexiu District, Guangzhou, 510080 China

**Keywords:** Trigeminal nerve angulation, Multiplanar reconstruction, Trigeminal neuralgia, Magnetic resonance imaging, Trigger maneuvers

## Abstract

**Objectives:**

Neurovascular compression (NVC) produces morphological changes on the trigeminal nerve root is considered the cause of trigeminal neuralgia (TN), but there were some patients with TN found no NVC, and also NVC was found in asymptomatic individuals. Many studies found tight relationships of TN and morphological structures of trigeminal nerve. We designed this study to explore the correlation between multiplanar reconstruction (MPR) trigeminal nerve angulation (TNA) and TN.

**Methods:**

Patients with classical symptoms of TN were recruited as observation group (OG) in this study, 50 healthy controls were enrolled as control group (CG), the OG was further subtyped into young patients (YP), middle-aged patients (MP) and old patients (OP) based to the onset age of symptoms, and also divided into patients with or without trigger maneuvers (TM) based on the presence of TM or not. All the participants underwent magnetic resonance (MR) examinations in same device, bilateral TNA measurements were carried out in OG and CG, then TNA was compared between different groups or subgroups. All images were interpreted by two radiologists who were blinded to the study, diagnosis of TN was made by two senior neurosurgery professors.

**Result:**

Ultimately, 95 patients with primary TN were recruited in OG, aged from 25 to 84 (61.15 ± 12.70) years with a course of 0.5 to 30 (5.03 ± 5.41) years, their onset age ranged from 24 to 82 (56.13 ± 11.98) years. There were 34 males and 61 females in OG, and 58 cases involved right side. The CG aged from 22 to 85 (61.86 ± 13.03) years. No statistical difference was found between the age of OG and CG(*p* = 0.76), and also the bilateral TNA of CG (154.92 ± 16.90° vs 155.55 ± 17.03°, *p* > 0.05), while TNA of OG was significantly smaller than CG (150.78 ± 11.29° vs 155.24 ± 16.88°, *p* = 0.019). In OG, TNA on the affected side was significantly smaller than the unaffected side (149.29 ± 12.44° vs 152.27 ± 9.85°, *p* = 0.014). TNA showed a positive correlation with onset age of patients with TN, as TNA on the affected side of YP was significantly smaller than MP and OP (139.00 ± 11.64° vs 148.86 ± 11.54°, 139.00 ± 11.64° vs 152.18 ± 12.61°, *p* = 0.004 and 0.026). Furthermore, patients with TM showed smaller TNA than those without TM (147.05 ± 11.30° vs 164.75 ± 8.39°, *p* < 0.001).

**Conclusions:**

This study suggested that TNA might play a role in TN, small TNA could be a risk factor of TN. Furthermore, patients with small TNA are more likely to combine with TM, but more studies are needed to explore the exact role of TNA in TN.

## Introduction

Trigeminal neuralgia (TN) is a common neuropathic pain mainly characterized by unilaterally paroxysmal and severe pain restricted in the distributions of trigeminal nerve. TN, which affects more in women and mainly affects right side, could lead to severe neuropsychiatric symptoms and badly affect life quality of patients [1–3]. TN is a relatively common disease in department of neurosurgery, with an age-associated prevalence of12.6–28.9/100000, about 70% of patients with TN are over 60 years old [1–3]. Of note, more than 90% of patients with TN might complain of trigger maneuvers (TM) of pain, among which touching, speaking, washing and eating are the most common [1, 4–6]. The International Association for the Study of Pain and the International Headache Society classified the TN primary TN and secondary TN, and primary TN could be further divided into idiopathic TN, classical TN [1]. Presently, NVC is the most accepted etiology of primary TN, but there were many patients with TN found no NVC, and NVC could be found in some asymptomatic individuals [7].

Diagnosis of TN mainly bases on clinical manifestations and images examinations [1, 8, 9]. Conventional magnetic resonance (MR) can identify neurovascular relationships and secondary TN, but it can’t clearly depict some small vessels for the thick layers [10]. Advanced sequences of MR could identify responsible vessels (RV) and neurovascular relationships of TN [11, 12], and studies focused on the relationships between TN features and morphology of trigeminal nerve, such as cerebellopontine angle (CPA) cistern cross-sectional area [13–16], coronal trigeminal sectional area and trigeminal atrophy [17–19]. Current study aimed to explore the correlation between TNA and TN.

## Methods and materials

From May 2020 to November 2021, patients with classical symptoms of TN (observation group, OG) and 50 healthy controls for physical examination (control group, CG) underwent MRI examinations in same device (Siemens, Erlangen, Germany), definite diagnosis of primary TN was made by two senior neurosurgery professors based on their clinical manifestations and neuroradiological features. The diagnosis of TN was accordingly with the third edition International Classification of Headache Disorders [1], basic data and neuroimaging data were collected in detail.

The inclusion criteria included 1). Primary TN was definitely diagnosed in OG [1, 8], and no positive results were found in CG. 2). All the patients underwent MR examinations in the same machine (Siemens, Erlangen, Germany). The exclusion criteria included 1). Participants with primary TN caused by vertebrobasilar artery or secondary TN [1]. 2). Patients combined with other cranial nerve diseases such as glossopharyngeal neuralgia or hemifacial spasm [20–22]. 3). Participant with cranial malformation that may affect the measurements of TN. 4). Patients took oral medications to control the symptoms, as oral medications might influence the presentation of TM. 5). The trigeminal nerve can’t be fully displayed and the TNA can’t be measured correctly.

All participants underwent high-resolution T2-weighted axial sequences include three-dimensional double echo steady-state interference sequence (3D-CISS) examinations with specific parameters (TR 1000 ms, TE 139 ms, matrix 384 × 384 mm, thickness 0.5 mm, slice number 56, flip angle 120°, field of view 200 mm) to include bilateral trigeminal nerve and guaranteed optimal display of trigeminal nerve. The sequences include coronal SE T1WI, TSE T2WI and FLIAR sequence, sagittal SE T2WI and 3D-CISS sequence. All the image data were measured by two radiologists who were blinded to the study.

**Fig. 1:**
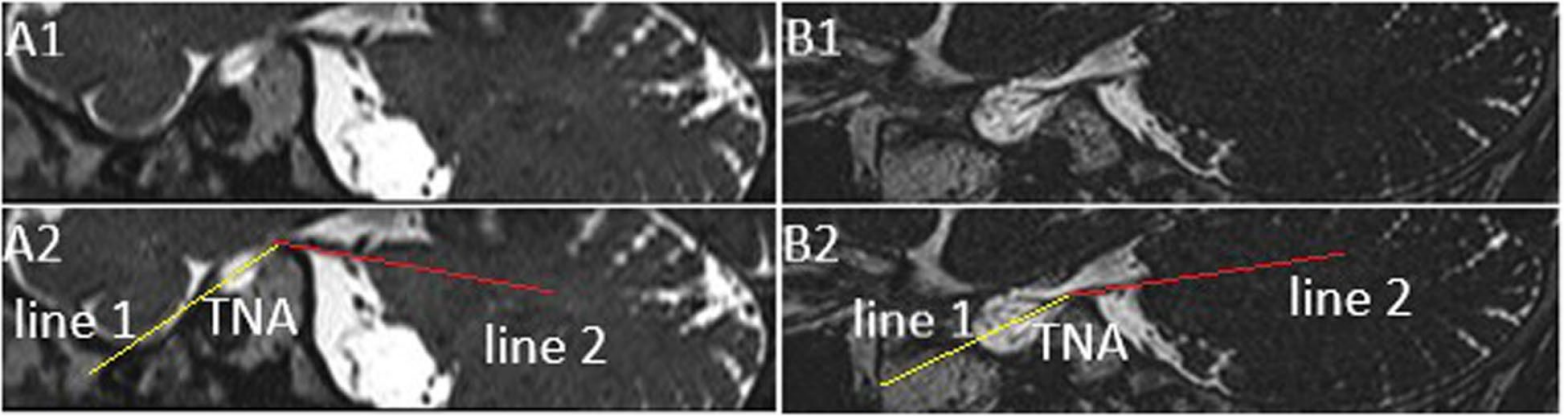
The multiplanar reconstruction of original magnetic resonance image data, 3D-T2-weighted images were reconstructed in the obliquely sagittal plane. The angle between line 1 and line 2 was considered as trigeminal nerve angulation (A2, B2). Color lines represented corresponding lines (yellow: line 1; red: line 2). Line 1: The obliquely sagittal image of trigeminal ganglion (A1-A2). If there were two or more images of the trigeminal branches, the line 1 was the image of the caudal branch of the trigeminal nerve (B1-B2). Line 2: The obliquely sagittal image of the trigeminal nerve on cerebellopontine angle cistern (A2, B2)

The multiplanar reconstruction (MPR) of 3D-CISS data was utilized on the workstation (sungo.via 2.0) to facilitate the measurement of the bilateral TNA. Obliquely sagittal plane which was centered on the long axis of trigeminal nerve was obtained at the outlet of Meckel’s cavity, and TNA was measured at the petrosal ridge, the angle between the line 1 and line 2 was defined as TNA. Line 1: The obliquely sagittal image of trigeminal ganglion. When there were two or three images of the trigeminal branches, the caudal one was used in the line 1. Line 2: The obliquely sagittal image of the nerve on CPA cistern (Fig. 1).

**Fig. 2:**
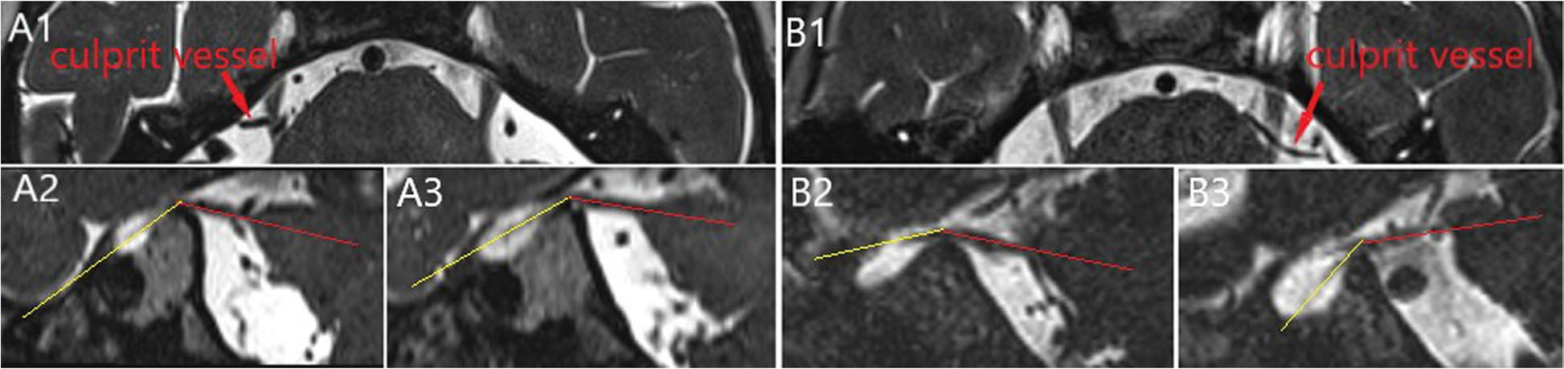
The measurements processes performed on two patients with trigeminal neuralgia. A1-A3 represented the measurements of bilateral trigeminal nerve angulation of a patient with right trigeminal neuralgia (A1), and the left trigeminal nerve angulation (A3) was larger than right trigeminal nerve angulation (A2). B1-B3 represented the measurements of bilateral trigeminal nerve angulation of a patient with left trigeminal neuralgia (B1), and the right trigeminal nerve angulation (B2) was larger than left trigeminal nerve angulation (B3)

The measurements of two patients with TN were showed in Fig. 2. Bilateral TNA comparisons of CG were used to assess the validity of the TNA comparison between OG and CG, then TNA was compared between OG and CG, affected and unaffected side of OG. The OG was further divided into young patients (YP) (< 40 years old), middle-aged patients (MP) (40–59 years old) and old patients (OP) (≥ 60 years old), the differences of TNA among the three subtypes were further compared. Based on the presence of TM or not, patients were also divided into two subgroups, differences of TNA between the two subgroups were also analyzed.

Statistical analysis was performed with SPSS software 26.0, the significance level was *p* < 0.05. The intraclass correlation coefficient (ICC) was used to evaluate the inter-radiologist reliability. ICC > 0.75 means excellent consistency. 0.4 ≤ ICC ≤ 0.75 means good consistency; ICC < 0.4 means poor consistency. The TNA on both sides of OG and CG (left and right sides in CG, affected and unaffected side in OG) were compared by the t test for paired samples. Age, duration of symptoms and onset age, TNA of patients with or without TM, TNA of OG and CG were compared by the t test for unpaired samples. One-Way ANOVA was used to test the TNA differences on affected and unaffected side among YP, MP and OP. Fisher’s exact test was used for the categorical data.

## Results

### Demographical features of the participants

Ultimately, 95 patients with TN, aged from 25 to 84 (61.15 ± 12.70) years, were enrolled in this study, course of TN ranged from 0.5 to 30 (5.03 ± 5.41) years, while onset age ranged from 24 to 82 (56.13 ± 11.98) years. There were 34 (35.79%) males and 61 (64.21%) females, while 58 (61.05%) patients affected the right side and 24 (25.26%) patients suffered from hypertension. The YP, MP and OP included 9(9.47%), 47(49.47%) and 39(41.05%) patients, respectively. 4(4.21%) patients affected the first branch of trigeminal nerve, 45(47.37%) patients affected the second branch of trigeminal nerve, 14(14.74%) patients affected the third branch of trigeminal nerve, 5(5.26%) patients affected the first and second branches of trigeminal nerve simultaneously, 25(26.32%) patients affected the second and third branches of trigeminal nerve simultaneously, 2(2.11%) patients affected the three branched of trigeminal nerve simultaneously. The courses of disease of 56 (58.95%) patients (58.95%) were less than 5 years, and courses of disease of 26 (27.37%) patients were between 5 and 9 years, and course of disease of 13 (13.68%) patients were no less than 10 years (Table 1). The CG included 26 (52%) females and 24 (48%) males with an age from 22 to 85 (61.86 ± 13.03) years, and 13 (26%) of them suffered from hypertension. Clinical data, including age, gender, course of disease, pain distribution, TM, hypertension and so on, were recorded, no significant differences in term of age, gender difference and hypertension incidence were found between the CG and OG (*p* > 0.05) (Table 2).

**Table 1:** Basic characteristics of the patients enrolled in this study(*n* = 95)

Characteristics	n (%)	Characteristics	n (%)
Gender		affected side	
Male	34(35.79)	Left	37(38.95)
Female	61(64.21)	Right	58(61.05)
Onset age(Y)		duration(Y)	
< 40	9(9.47)	< 5	56(58.95)
40–59	47(49.47)	5–9	26(27.37)
≥ 60	39(41.05)	≥ 10	13(13.68)
Branches		trigger maneuvers	
V 1	4(4.21)	no	12(12.63)
V2	45(47.37)	yes	83(87.37)
V3	14(14.74)	touching^a^	64(77.11)
V1 + V2	5(5.26)	eating^a^	42(50.60)
V2 + V3	25(26.32)	talking^a^	41(49.40)
V1 + V2 + V3	2(2.11)	brushing^a^	31(37.35)

^a^ The total percentage of trigger maneuvers was more than 100% because patients might experience more than one trigger maneuvers

*TN* Trigeminal neuralgia, *Y* Years old/year, *V1* Ophthalmic division, *V2* Maxillary division, *V3* Mandibular division

**Table 2:** Comparisons of basic features between observation group and control group

Characteristics	Observation group(*n* = 95)	Control group(*n* = 50)	*P* value
Age(Y)	61.15 ± 12.70	61.86 ± 13.03	0.76
Gender(F/M)	34/61	24/26	0.15
Hypertension	24	13	0.92
Trigeminal nerve angulation^a^	150.78 ± 11.29°	155.24 ± 16.88°	0.019*

^a^ Trigeminal nerve angulation was the mean of the bilateral side of observation and control group, respectively

*Y* Years old, *F* Female, *M* Male

^*^ *p* < 0.05

### TNA comparisons of OG and CG

As for the measurements of TNA, an ICC more than 0.90 (*p* < 0.001) was obtained from the two radiologists. The mean TNA in OG was significantly smaller than CG (150.78 ± 11.29° VS 155.24 ± 16.88°, *p* = 0.019), and no statistic difference was found of the TNA between the left and right sides of CG (154.92 ± 16.90° vs 155.55 ± 17.03°, *p* = 0.800) (Table 2).

### TNA comparisons on the affected and unaffected sides of OG

TNA on the affected side of OG was significantly smaller than the unaffected side (149.29 ± 12.44° vs 152.27 ± 9.85°, *p* = 0.014). TNA on the affected side of YP was significantly smaller than the unaffected side (139.00 ± 11.64° vs 147.22 ± 7.98°, *p* = 0.006). In the MP, no significant difference was found between the TNA on the affected and unaffected sides (148.86 ± 11.54° vs 150.15 ± 8.91°, *p* = 0.496). TNA on the affected side of OP was significantly smaller than the unaffected side (152.18 ± 12.61° vs 156.00 ± 10.25°, *p* = 0.036) (Table 3).

**Table 3:** Comparisons of trigeminal nerve angulation between affected and unaffected side of different age cohorts in observation group

Group	Affected side	Unaffected side	*P* value
Overall	149.29 ± 12.44°	152.27 ± 9.85°	0.014*
YP	139.00 ± 11.64°	147.22 ± 7.98°	0.006*
MP	148.86 ± 11.54°	150.15 ± 8.91°	0.496
OP	152.18 ± 12.61°	156.00 ± 10.25°	0.031*

*YP* Young patients, *MP* Middle-aged patients, *OP* Old patients

^*^ *p* < 0.05

### TNA comparisons of different age groups

Most of the 95 OG were MP (*n* = 47, 49.47%) and OP (*n* = 39, 41.05%) with a mean age of 51.28 ± 5.10 years and 67.41 ± 5.27 years, the mean age of YP was 32.56 ± 4.64 years. Bilateral TNA increased with age in three subgroups, and TNA showed great difference among different age groups. TNA were 152.18 ± 12.61° in OP, 148.86 ± 11.54° in MP and 139.00 ± 11.64° in YP on the affected side, and 156.00 ± 10.25° in OP, 150.15 ± 8.91° in MP and 147.22 ± 7.98° in YP on the unaffected side. Statistical analysis showed that the TNA in YP were significantly smaller than OP and MP on the affected side (*p* = 0.004; 0.026), TNA in OP were significantly larger than MP and YP on the unaffected side (*p* = 0.005, 0.013). However, no significant differences were obtained between the affected side of MP and OP, and also unaffected side of YP and MP (*p* = 0.205, 0.396) (Tables 4 and 5).

**Table 4:** One-Way ANOVA study of TNA on affected and unaffected side among different age groups in observation group

side	YP	MP	OP	F	*p*
affected side	139.00°±7.98°	148.86°±11.54°	152.18°±12.61°	4.47	0.014*
unaffected side	147.22°±7.98°	150.15°±8.91°	156.00±10.25°	5.55	0.005*

*TNA* trigeminal nerve angulation, *YP* young patients, *MP* middle-aged patients, *OP* old patients

*: *p* < 0.05

**Table 5:** Post hoc multiple comparisons of the three age groups on affected and unaffected sides in observation group

Side	Group	Group	TNA	Mean difference (95% CI)	*P* value
Unaffected side	YP (147.22° ± 7.98°)	MP	150.15° ± 8.91°	-2.92° ± 3.42°(-9.71°, 3.88°)	0.40
		OP	156.00 ± 10.25°	-8.78°3.49° (-15.69, -1.87)	0.013*
	MP (150.15° ± 8.91°)	YP	147.22° ± 7.98°	2.92° ± 3.42°(- 3.88°, 9.71°)	0.40
		OP	156.00 ± 10.25°	-5.86° ± 2.04° (-9.91°, -1.82°)	0.005*
	OP (156.00 ± 10.25°)	YP	147.22° ± 7.98°	8.78° ± 3.49° (1.87°, 15.69°)	0.013*
		MP	150.15° ± 8.91°	5.86° ± 2.04° (1.82°,9.91°)	0.005*
Affected side	YP (139.00° ± 7.98°)	MP	148.86° ± 11.54°	-9.86° ± 4.37° (-18.53°, -1.19°)	0.026*
		OP	152.18° ± 12.61°	-13.18° ± 4.44° (-21.99°, -4.37°)	0.004*
	MP (148.86° ± 11.54°)	YP	139.00° ± 7.98°	9.86° ± 4.37° (1.19°, 18.53°)	0.026*
		OP	152.18° ± 12.61°	-3.32° ± 2.60° (-8.48°, 1.85°)	0.205
	OP (152.18° ± 12.61°)	YP	139.00° ± 7.98°	13.18° ± 4.44° (4.37°, 21.99°)	0.004*
		MP	148.86° ± 11.54°	3.32° ± 2.60° (-1.85°, 8.48°)	0.205

*TNA* Trigeminal nerve angulation, *YP* Young patients, *MP* Middle-aged patients, *OP* Old patients, *CI* Confidence interval

^*^ *p* < 0.05

### Trigger maneuvers and TNA

In current study, 83 (87.37%) of patients complained of pain for mild stimuli, touching (77.11%), eating (50.60%), speaking (49.40%), and washing (37.35%) were the most common (Table 1). The TNA on the affected side of patients with TM was significantly smaller than those without TM (147.05 ± 11.30° vs 164.75 ± 8.39°, *p* < 0.001) (Table 6).

**Table 6:** Characteristics comparisons of patients with or without trigger maneuvers

Characteristics	with trigger maneuvers (*n* = 83)	without trigger maneuvers (*n* = 12)	*P* value
onset age(Y)	55.24 ± 12.27	62.25 ± 7.61	0.058
duration(Y)	5.01 ± 5.00	5.13 ± 8.00	0.95
age(Y)	60.25 ± 13.09	67.38 ± 7.35	0.011*
gender(F/M)	53/30	7/5	0.96
affected side(R/L)	50/33	8/4	0.91
hypertension	19	5	0.29
trigeminal nerve angulation on unaffected side (°)	151.16 ± 8.97	159.99 ± 12.51	< 0.001*
trigeminal nerve angulation on affected side (°)	147.05 ± 11.30	164.75 ± 8.39	0.003*

*Y* Years old/year, *F* Female, *M* Male, *R* Right side, *L* Left side

^*^ *p* < 0.05

## Discussions

As a common pain syndrome, TN causes lots of uncomfortableness, even severe psychiatric symptoms [1–3]. With the sound developments of image and surgical techniques, the diagnosis and treatments of TN get great progress. NVC is nowadays widely accepted mechanism of TN, and the presence of symptoms and imagine examinations is the diagnostic criteria of classical TN [1, 2, 4, 8]. However, there are many asymptomatic individuals with NVC [23, 24], some other factors may play a role in the presence of TN.

### Value of MR in diagnosis of TN

MR imagine tests play vital roles in diagnosis of TN. Hung et al. found that diffusion tensor imaging (DTI) could assist the diagnosis of TN [25], positive correlations between nerve atrophy, small trigeminal pontine angle, small CPA cistern cross-sectional area, short trigeminal nerve, thin trigeminal nerve with TN had been demonstrated [13–16, 19, 26, 27]. Sheng Cheng Wei et al. reported that three-dimensional time-of-flight (3D-TOF) MR angiography imaging showed a sensitivity rate (SR) of 74.0% in the evaluation of NVC, the SR could reach to 88.4% when combined with 3D-CISS [11]. H G Boecher-Schwarz et al. reported an 88.9% of positive rate (PR) and 88.5% of SR of NVC detection from high-definition MR angiography [12]. Andrei Brînzeu [28] even found 3D-MR imaging could definite 97% of NVC. Similar results could also be reached with 3D-SPACE, Hong Duc Pham et al. [29] found that fused 3D-T2-SPACE plus 3D-TOF MR angiography imaging was an effectively tools for treatment planning of TN, the SR of NVC evaluation could reach 94%. Here, we make a brief summary of the advantages and disadvantages of different MR sequences in the display of trigeminal root entry zone and adjacent vessels (Table 7).

**Table 7:** Advantages and disadvantages of different magnetic resonance sequences for trigeminal root entry zone and adjacent vessels

Sequence	Advantages	Disadvantages
3D-TOF-MR	Relationship between artery and nerve is clearly displayed	Poor visualization of low flow and small vessels
3D-CISS	Coronal and oblique sagittal reconstruction to visualize the vessel and its origination surrounding the nerve	Low contrast ratio to soft tissue, even between soft tissue and bone
3D-FIESTA	Hyperintense cerebrospinal fluid is clearly contrasted with blood vessels and hypointense nerves	Low contrast resolution between soft and hard tissues
3D-SPACE	High contrast ratio between cerebrospinal fluid and tissues, providing accurate visualization of the neurovascular structures	High T2WI signal impact the display of small vessel

*3D-TOF-MR* Three-dimensional time-of-flight magnetic resonance, *3D-CISS* Three-dimensional double echo steady-state interference sequence

MR could predict prognosis of primary TN. Obata et al.found that smaller preoperative cistern deviation index was positively correlated to the recurrence of TN after microvascular decompression (MVD) [14]. What’s more, Andrei Brînzeu et al. [28] and Patrick W Hitchon et al. [30] found degree of NVC on MR could accurately predict the outcomes of patients with TN who underwent MVD. Coincidentally, Matthew S Willsey et al. [31] pointed out pontine-segment diffusion tensor MR radial diffusivity (RD) could accurately predict the outcomes of TN after MVD.

### Correlations of trigeminal morphology with pathogenesis and manifestations of TN

Trigeminal morphological studies could prompt the mechanisms, diagnoses study of TN. Neuroanatomical studies revealed that trigeminal nerve would turn as it passed through the petrosal ridge, Andrei Brinzeu et al. [32] found petrosal ridge played a nonnegligible role in TN, they thought it was a potential compressional point of trigeminal nerve, which may prompt the occurrence of TN, especially in small CPA cistern or severe hemisphere atrophy cases [16]. In such case, the nerve was stretched, brain stem and cerebellum may ptosis, which was commonly known as *sagging phenomena*. These factors prompt the formation of NVC [16, 27, 30, 33, 34].

It’s reported that prevalence of TN increased with age [35], and the average onset age was 53–57 years [36, 37], and about 70% of patients with TN are over 60 years old [1–3]. We found that the TNA on the affected side was significantly smaller than the unaffected side, the TNA increased with the onset age of patients with TN, and TNA on the affected side of YP was significantly smaller than that of OP and MP, which suggested that TNA was related to the onset age of TN and small TNA might be a risk factor of TN.

Mild stimuli induced pain is one of the basic characteristics of TN, a great proportion of patients with TN complained of TM [1, 4]. Antiepileptic drugs, such as carbamazepine and oxcarbazepine, were regarded as the first-line choice for TN, almost 90% of patients could get pain control [8]. As oral medications might influence TM, patients with a history of oral medications before were excluded in this study. We found most (87.37%) patients complained of TM, including touching, eating, speaking and washing, it was consistent with previous studies [1, 4–6]. P Ferroli et al. held that compression of trigeminal nerve root led to nerve demyelination, affected impulse function of trigeminal nerve and activated negative feedback regulation, then the nerve get hypersensitive, even mild stimulations could trigger impulses [38]. Andrei Brinzeu and Sami H Erbay et al. found that certain morphological changes, including axonal loss, axonopathy, demyelination, residual myelin debris, and collagen deposition, can occur following long-term stress on trigeminal nerve [32, 39], these factors might prompt the presence of TM. We found small TNA in patients with TN was closely related to the presence of TM, but the exact mechanism needs to be further explored.

### Limitations

There were some limitations in current study. Firstly, all of the subjects were from one single center and may be biased. Furthermore, the evaluation of TNA was limited by the sample.

## Conclusions

The results of current study suggested that TNA might play a role in TN, TNA on patients with TN and common people showed significant difference, and statistic difference was found between the affected and unaffected side of patients with TN, which indicated that small TNA could be a risk factor of TN. Moreover, patients with small TNA are more likely to combine with TM, but more studies are needed to explore the exact role of TNA in TN.

## Data Availability

All data are fully available without restriction by contacting Tao Sun or Chao Yang.

## Abbreviations

MPR: Multiplanar reconstruction
TNA: Trigeminal nerve angulation
TN: Trigeminal neuralgia
OG: Observation group
CG: Control group
YP: Young patients
MP: Middle-aged patients
TM: Trigger maneuvers
OP: Old patients
MR: Magnetic resonance
NVC: Neurovascular compression
3D-TOF: Three-dimensional time-of-flight
3D-CISS: Three-dimensional double echo steady-state interference sequence
RV: Responsible vessels
SR: Sensitivity rate
PR: Positive rate
RD: Radial diffusivity
SCA: Superior cerebellar artery
CPA: Cerebellopontine angle
MVD: Microvascular decompression
